# Prediction of Hepatocellular Carcinoma Development after Hepatitis C Virus Eradication Using Serum *Wisteria floribunda* Agglutinin-Positive Mac-2-Binding Protein

**DOI:** 10.3390/ijms17122143

**Published:** 2016-12-20

**Authors:** Shunsuke Sato, Takuya Genda, Takafumi Ichida, Nozomi Amano, Sho Sato, Ayato Murata, Hironori Tsuzura, Yutaka Narita, Yoshio Kanemitsu, Katsuharu Hirano, Yuji Shimada, Katsuyori Iijima, Ryo Wada, Akihito Nagahara, Sumio Watanabe

**Affiliations:** 1 Department of Gastroenterology and Hepatology, Juntendo University Shizuoka Hospital, Shizuoka 410-2295, Japan; syusato@juntendo.ac.jp (S.S.); n-amano@juntendo.ac.jp (N.A.); sho-sato@juntendo.ac.jp (S.S.); perseverance3@hotmail.co.jp (A.M.); htudura@juntendo.ac.jp (H.T.); ynarita@juntendo.ac.jp (Y.N.); yoshio-k.s52@live.jp (Y.K.); yshimada@juntendo.ac.jp (Y.S.); katsu0716@shore.ocn.ne.jp (K.I.); nagahara@juntendo.ac.jp (A.N.); 2 Department of Hepatology, East Shonan General Hospital, Kanagawa 253-0083, Japan; takafumi@air.ocn.ne.jp (T.I.); khirano-tym@umin.ac.jp (K.H.); 3 Department of Pathology, Juntendo University Shizuoka Hospital, Shizuoka 410-2295, Japan; mdksmrwjunten522@ybb.ne.jp; 4 Department of Gastroenterology, Juntendo University School of Medicine, Tokyo 113-8431, Japan; sumio@juntendo.ac.jp

**Keywords:** WFA^+^-M2BP, hepatocellular carcinoma, chronic hepatitis C, risk factor, sustained virological response

## Abstract

We aimed to clarify the association between a novel serum fibrosis marker, *Wisteria floribunda* agglutinin-positive Mac-2-binding protein (WFA^+^-M2BP), and hepatocellular carcinoma (HCC) development in 355 patients with chronic hepatitis C who achieved sustained virologic response (SVR) through interferon-based antiviral therapy. Pretreatment serum WFA^+^-M2BP levels were quantified and the hazard ratios (HRs) for HCC development were retrospectively analyzed by Cox proportional hazard analysis. During the median follow-up time of 2.9 years, 12 patients developed HCC. Multivariate analysis demonstrated that high serum WFA^+^-M2BP (≥2.80 cut off index (COI), HR = 15.20, *p* = 0.013) and high fibrosis-4 (FIB-4) index (≥3.7, HR = 5.62, *p* = 0.034) were independent risk factors for HCC development. The three- and five-year cumulative incidence of HCC in patients with low WFA^+^-M2BP were 0.4% and 0.4%, respectively, whereas those of patients with high WFA^+^-M2BP were 7.7% and 17.6%, respectively (*p* < 0.001). In addition, combination of serum WFA^+^-M2BP and FIB-4 indices successfully stratified the risk of HCC: the five-year cumulative incidences of HCC were 26.9%, 6.8%, and 0.0% in patients with both, either, and none of these risk factors, respectively (*p* < 0.001). In conclusion, pretreatment serum WFA^+^-M2BP level is a useful predictor for HCC development after achieving SVR.

## 1. Introduction

Chronic hepatitis C virus (HCV) infection frequently causes liver cirrhosis and hepatocellular carcinoma (HCC) development. HCC is currently one of the most common cancers and cause of cancer-related death worldwide [1], with chronic HCV infection being responsible for many HCC cases in Europe and the United States as well as in Japan [2,3]. Notably, the risk of HCC development is 20-fold higher in patients with HCV as compared with those without HCV [4], indicating a significant carcinogenic role of chronic HCV infection. Therefore, chronic hepatitis C (CHC) patients have been widely treated with interferon-based antiviral therapy because the treatment not only eradicates HCV, but also reduces the risk of HCC development. The risk of developing HCC is most effectively lowered in patients who achieve a sustained virologic response (SVR) by interferon therapy [5,6,7], and recent advances of direct-acting antiviral drugs (DAAs) against HCV have improved the SVR rate of interferon therapy [8,9]. Additionally, the number of patients receiving antiviral therapy are now increasing because of the safety of the all-oral combination therapy associated with DAAs [10,11]. As a result, a large number of CHC patients achieve SVR in current real-world clinical settings; however, the risk of HCC development remains even in patients who achieve SVR [12]. Therefore, the necessity of useful predictors for HCC development in SVR patients becomes high in the management of patients with CHC.

Factors such as older age, male gender, and hepatic fibrosis, have been reported to predict the risk of HCC development in patients with CHC [13,14]. Specifically, the existence of advanced hepatic fibrosis or cirrhosis prior to treatment is widely recognized as an important risk factor for HCC development, even after achieving SVR [15]. Liver biopsy has been the gold standard for assessment of liver fibrosis [16], although there might be a staging misdiagnosis based on sampling errors and intra- and inter-observer variability [17,18]. Moreover, its invasiveness and rare, but potentially life-threatening, complication make it difficult to perform liver biopsy for all patients [16]. As a result, several alternative laboratory liver fibrosis indices have been proposed, i.e., aspartate aminotransferase (AST)-to-platelet ratio index (APRI) and fibrosis-4 (FIB-4) index [19,20]. Recently, a new glycol marker for liver fibrosis was developed using the glycan sugar chain-based immunoassay. *Wisteria floribunda* agglutinin-positive Mac-2-binding protein (WFA^+^-M2BP) was identified as a fibrosis-related glycol-alteration [21], and a significant association between its serum levels and histological hepatic fibrosis was reported in chronic liver diseases [22]. WFA^+^-M2BP level can be quantified in a small amount of serum samples (10 µL) using an automatic and high throughput assay [21]. This advantage over liver biopsy suggests the clinical usefulness of WFA^+^-M2BP for assessing the risk of HCC development. Here, we evaluated factors that affect the occurrence of HCC in patients with CHC achieving SVR through interferon-based antiviral therapy, with a special focus on the predictive value of serum WFA^+^-M2BP levels.

## 2. Results

### 2.1. Patients’ Characteristics and Hepatocellular Carcinoma (HCC) Development after Sustained Virologic Response (SVR)

Table 1 summarizes the profiles and laboratory data of the 355 patients enrolled in this study. Pegylated interferon plus ribavirin combination therapy for 24 to 72 weeks was administrated to 299 of the 355 patients. Protease inhibitor treatment together with pegylated interferon plus ribavirin for 24 weeks was administrated to 56 patients (25 telaprevir, 22 simeprevir, and 9 faldaprevir). Of the 355 patients, 12 (3.4%) developed HCC during a median follow-up of 2.9 years (range, 0.5–9.6 years). The estimated cumulative incidences of HCC development in the entire cohort were 2.4% and 5.4% at three and five years, respectively. Compared with patients who did not develop HCC, those who developed HCC were older (*p* = 0.005) and exhibited a higher degree of histological fibrosis (*p* < 0.001) and inflammation (*p* = 0.018), lower albumin levels (*p* < 0.001), lower platelet counts (*p* < 0.001), and higher α-fetoprotein (AFP) levels (*p* < 0.001). Both APRI and FIB-4 index were significantly higher in patients with HCC than in patients without HCC.

### 2.2. Serum WFA^+^-M2BP Levels in the Study Cohort

**Figure 1:**
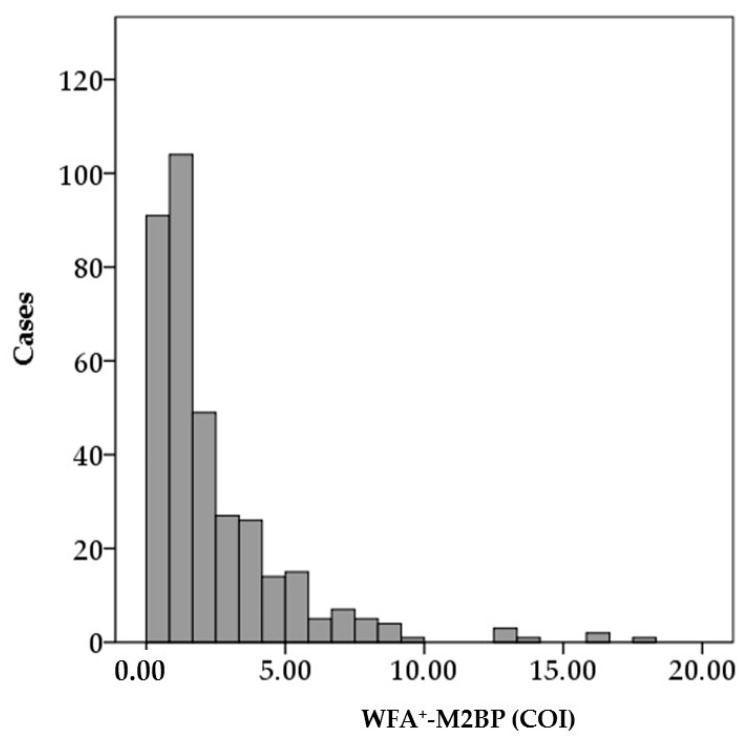
Distribution of serum *Wisteria floribunda* agglutinin-positive Mac-2-binding protein (WFA^+^-M2BP) levels in the study cohort. COI, cut off index.

**Figure 2:**
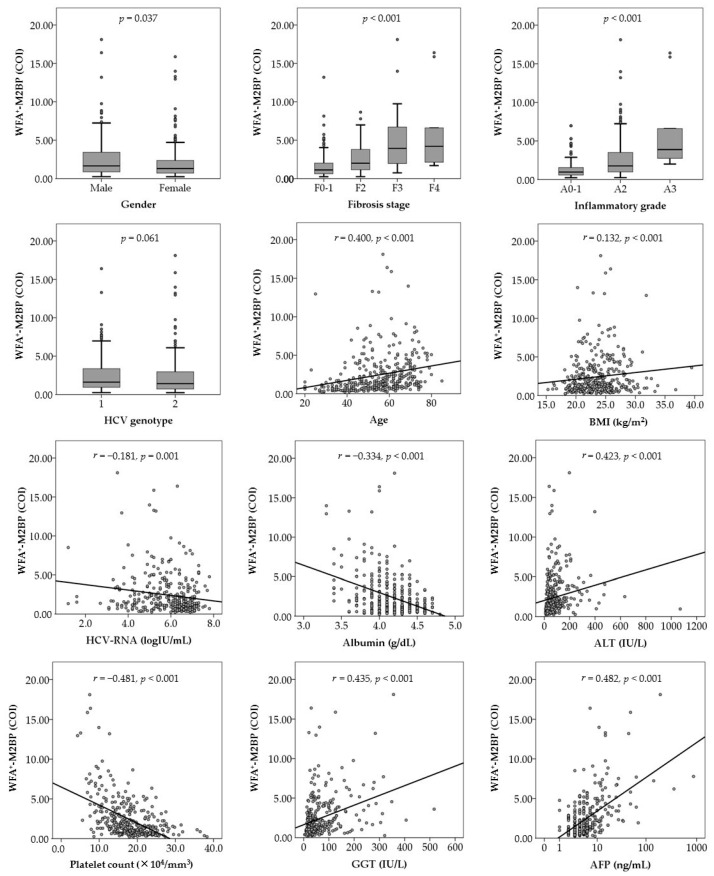
Relationships between *Wisteria floribunda* agglutinin-positive Mac-2-binding protein (WFA^+^-M2BP) levels and patient characteristics at baseline. The Mann–Whitney *U* test or Kruskal–Wallis test was used for the categorical data. Spearman’s rank correlation coefficient was used for the continuous data. AFP, α-fetoprotein; ALT, alanine aminotransferase; BMI, body mass index; GGT, γ-glutamyl transpeptidase; HCV, hepatitis C virus.

Figure 1 shows the distribution of serum WFA^+^-M2BP levels in the study cohort. The median WFA^+^-M2BP level was 1.47 (range, 0.23–18.11). Relationships between serum WFA^+^-M2BP levels and baseline patients’ characteristics are shown in Figure 2. Serum WFA^+^-M2BP levels were significantly higher in males than in females, in patients with advanced hepatic fibrosis than in patients with mild hepatic fibrosis, and in patients with severe inflammation than in patients with mild inflammation. Serum WFA^+^-M2BP levels were positively correlated with the patients’ age, body mass index (BMI), alanine aminotransferase (ALT), γ-glutamyl transpeptidase (GGT), and AFP, and were negatively correlated with serum HCV-RNA, albumin, and platelet count (Figure 2).

### 2.3. Risk Analysis

**Figure 3:**
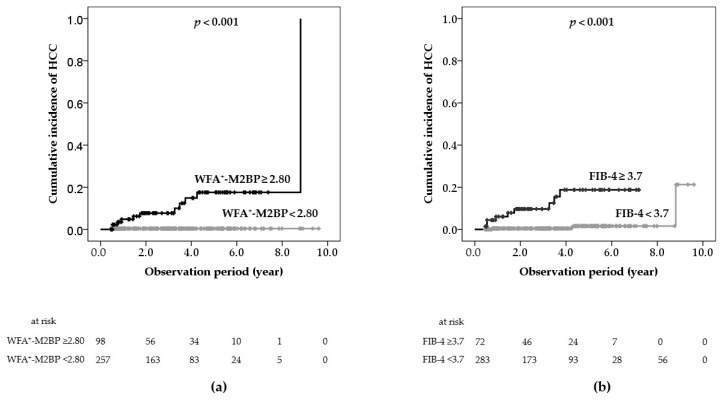
Cumulative incidence of hepatocellular carcinoma (HCC) development after sustained virologic response, shown according to serum *Wisteria floribunda* agglutinin-positive Mac-2-binding protein (WFA^+^-M2BP) level (**a**) or fibrosis-4 (FIB-4) index (**b**).

To identify non-invasive markers predicting HCC development in patients achieving SVR, Cox proportional hazard analysis was performed on five baseline patients’ characteristics, nine biochemical data, and two indices calculated from biochemical data (Table 2). Univariate analysis revealed that age (≥60 years), HCV-RNA (<6.0 logIU/mL), serum albumin (≤4.0 g/dL), GGT (≥55 IU/L), platelet count (≤8.0 × 10^4^/µL), AFP (≥8.0 ng/mL), FIB-4 index (≥3.7), APRI (≥1.25), and WFA^+^-M2BP (≥2.80 COI) were associated with HCC development. Multivariate analysis identified two independent risk factors: WFA^+^-M2BP level and FIB-4 index. The three- and five-year cumulative incidence rates of HCC development in patients with WFA^+^-M2BP <2.80 were 0.4% and 0.4%, respectively, whereas those of patients with WFA^+^-M2BP ≥2.80 were 7.7% and 17.6%, respectively (*p* < 0.001, Figure 3a). The cumulative incidence rates of HCC development in patients with FIB-4 index <3.7 were 0.4% and 1.6%, respectively, whereas those of patients with FIB-4 index ≥3.7 were 9.7% and 18.8%, respectively (*p* < 0.001, Figure 3b).

**Figure 4:**
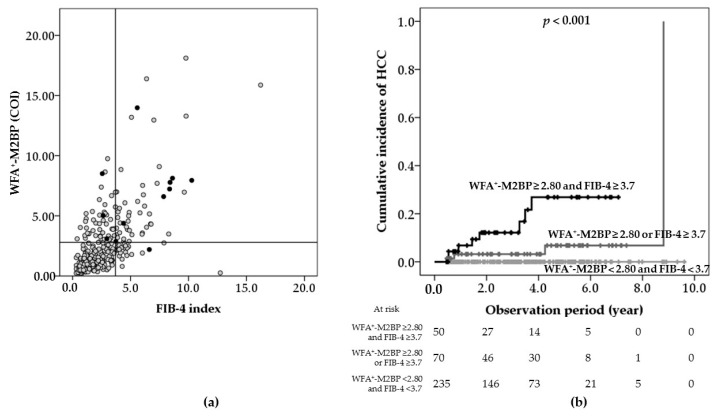
(**a**) Relationship between serum *Wisteria floribunda* agglutinin-positive Mac-2-binding protein (WFA^+^-M2BP) levels and Fibrosis-4 (FIB-4) index. Black and gray circles represent patients with and without HCC development, respectively. Vertical and horizontal line represent FIB-4 index = 3.7 and WFA^+^-M2BP = 2.80 COI, respectively; (**b**) Cumulative incidence of hepatocellular carcinoma (HCC) development after sustained virologic response, shown according to serum WFA^+^-M2BP level and FIB-4 index.

The number of these risk factors varied between patients. Fifty patients (14.1%) presented both risk factors (WFA^+^-M2BP ≥ 2.80 and FIB-4 index ≥ 3.7), 70 patients (19.7%) had either of these risk factors (WFA^+^-M2BP ≥ 2.80 or FIB-4 index ≥ 3.7), and the remaining 235 patients (66.2%) had none of these risk factors (WFA^+^-M2BP < 2.80 and FIB-4 index < 3.7) (Figure 4a). Patients without these risk factors did not develop HCC during the study period. In patients with either of these two risk factors, the cumulative incidence of HCC at three and five years were 3.2% and 6.8%, respectively. In patients with both of these two risk factors, the cumulative incidence of HCC at three and five years were 12.1% and 26.9%, respectively. The cumulative incidences of HCC were significantly different (log-rank test, *p* < 0.001) (Figure 4b).

### 2.4. Hepatic Fibrosis Stage and Serum WFA^+^-M2BP and FIB-4 Index

**Figure 5:**
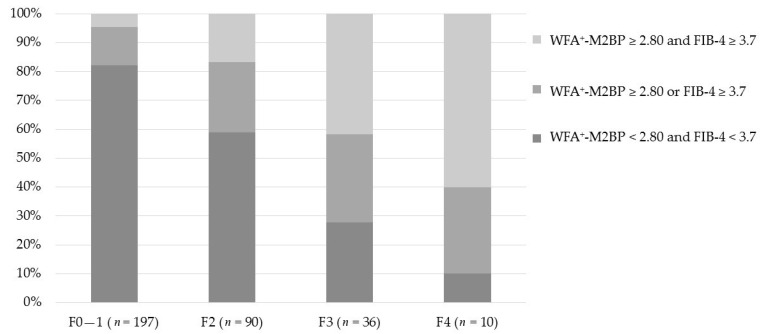
Serum *Wisteria floribunda* agglutinin-positive Mac-2-binding protein (WFA^+^-M2BP) level and Fibrosis-4 (FIB-4) index in each fibrosis stage. *n* = 333.

The proportion of patients with different numbers of risk factors stratified by the fibrosis stage was analyzed in 333 patients whose histological fibrosis stage information was available (Figure 5). The proportion of patients with both risk factors (WFA^+^-M2BP ≥ 2.80 and FIB-4 index ≥ 3.7) was 9 cases (4.6%) in F0–1, 15 cases (16.7%) in F2, 15 cases (41.7%) in F3, and 6 cases (60.0%) in F4. The proportion of patients with either risk factor (WFA^+^-M2BP ≥ 2.80 or FIB-4 index ≥ 3.7) was 26 cases (13.2%) in F0-1, 22 cases (24.4%) in F2, 11 cases (30.6%) in F3, and 3 cases (30.0%) in F4. There was a significant difference in the proportion of patients with different numbers of risk factors among fibrosis stages (*χ*^2^ test, *p* < 0.001).

## 3. Discussion

CHC patients with pre-existing liver cirrhosis or severe hepatic fibrosis have a higher risk of developing HCC [2], even after achieving SVR through interferon based antiviral therapy [12]. Liver cirrhosis can be easily diagnosed in cases showing signs of end-stage liver disease such as ascites, jaundice, variceal bleeding, and hepatic encephalopathy. However, diagnosis of cirrhosis is difficult if laboratory findings show normal or near normal levels in compensated stage. Liver biopsy has been considered the gold standard diagnostic method for the assessment of early compensated cirrhosis or advanced fibrosis, but it can exhibit sampling variability and risk of lethal complications as a potential limitation of liver biopsy [16,17,18]. In the present study, we identified high serum WFA^+^-M2BP, a new serum marker for liver fibrosis, as an independent risk factor for HCC development after achieving SVR. Serum WFA^+^-M2BP of ≥2.80 was associated with a >15-fold-increased risk of HCC development in patients who achieved SVR. The threshold ≥2.80 demonstrated in this study as a risk for HCC is a close value to the previously reported cutoff value of 2.64 for liver cirrhosis [22], further indicating pre-existing liver cirrhosis as a risk factor for HCC development after achieving SVR. Interestingly, a recent report demonstrated that the cutoff values for predicting cirrhosis and HCC were 1.26 and 0.71, respectively, in patients with chronic hepatitis B virus infection [23]. These observations suggest that the WFA^+^-M2BP cutoff value for predicting cirrhosis or HCC varies depending on the etiology.

WFA^+^-M2BP was originally reported as a fibrosis-related glycol-alteration, and a significant relationship between serum WFA^+^-M2BP level and histological fibrosis stage was confirmed in the present study. Platelet count and serum albumin level, which decreased in association with the progression of hepatic fibrosis [24,25], were associated with serum WFA^+^-M2BP level as well. Age is also known as a surrogate marker of disease duration and is associated with more advanced fibrosis [26]. In addition, other biochemical factors correlated with serum WFA^+^-M2BP level. ALT, GGT, and AFP, positively correlated with serum WFA^+^-M2BP level. ALT is a sensitive indicator of necroinflammatory activity in the liver, and AFP levels without HCC are also related to liver cell damage [27]. Serum GGT is a surrogate of oxidative stress [28]. These results suggest that serum WFA^+^-M2BP level is affected by not only hepatic fibrosis, but also by necroinflammatory activity or hepatocyte damage in the liver. Indeed, a report demonstrated that WFA^+^-M2BP as well as ALT levels decreased just after achieving HCV eradication [29]. Moreover, several demographic variables correlated with serum WFA^+^-M2BP level; higher age, male gender, and higher BMI were associated with higher serum WFA^+^-M2BP level. Interestingly, some of them were reported as risk factors for HCC development [30]. Thus, serum WFA^+^-M2BP level possibly reflects HCC risk other than hepatic fibrosis.

Similar to WFA^+^-M2BP, FIB-4 index was developed as a surrogate marker of liver fibrosis in patients with CHC [20], and its usefulness to predict HCC has been reported [31]. However, risk assessment by these two fibrosis markers showed some discrepancies in this study. WFA^+^-M2BP was sometimes high even in patients without high FIB-4 index, whereas other patients had high FIB-4 index without high serum WFA^+^-M2BP. Such patients are nevertheless at risk of HCC. In the histologically confirmed cirrhotic (F4) and advanced hepatic fibrosis (F3) patients, both high WFA^+^-M2BP and FIB-4 index were observed only in 60.0% and 41.7% of the patients, respectively. However, high WFA^+^-M2BP and/or FIB-4 index were observed in 90% and 72.3% of F4 and F3 patients, respectively. These results suggest that WFA^+^-M2BP and FIB-4 index predict advanced fibrosis or compensated cirrhosis in a complementary manner.

The present study demonstrates that a combination of serum WFA^+^-M2BP level and FIB-4 index could be used to stratify the risk of HCC. Patients with low WFA^+^-M2BP and FIB-4 index presented a very low risk of HCC development, and patients with either high WFA^+^-M2BP or high FIB-4 index had a moderate risk. Conversely, patients with both high WFA^+^-M2BP and high FIB-4 index had an extremely high risk of HCC even after achieving SVR. In clinical practice, the interval of HCC surveillance should be decided based on personal HCC risk. Indeed, either serum WFA^+^-M2BP level or FIB-4 index was previously demonstrated as a risk factor for developing HCC [31,32,33]. However, this study proposed a novel, non-invasive risk assessment based on the combination of WFA^+^-M2BP and FIB-4 index. Our data indicate that patients with WFA^+^-M2BP ≥2.80 and FIB-4 index ≥3.7 require the most intensive HCC surveillance even after achieving SVR.

The main limitation was that this study was retrospectively performed in a single center; therefore, the number of cases of HCC development might be small for the analysis. A future large-scale prospective analysis will be required to validate our results. In addition, a recent report demonstrated that some post-treatment biochemical parameters had higher predictive value for HCC development than pre-treatment parameters [34]. From this point of view, chronological change of serum WFA^+^-M2BP levels before and after anti-viral treatment and its association with HCC development are of interest and should be analyzed in a future study.

## 4. Materials and Methods

### 4.1. Patients

Between March 2004 and November 2014, 355 patients who achieved SVR by interferon-based anti-viral therapy were enrolled in this study at Juntendo University Shizuoka Hospital. All 355 patients met the following inclusion and exclusion criteria: (1) Diagnosis of chronic hepatitis C; (2) Negativity for hepatitis B surface antigen or human immunodeficiency virus; (3) Negative history of other chronic liver diseases (autoimmune hepatitis, primary biliary cirrhosis, hemochromatosis, and Wilson’s disease); (4) Absence of HCC or any suspicious lesions detected through ultrasonography, dynamic computed tomography, or magnetic resonance imaging at enrollment; (5) Negative history of previous treatment for HCC and liver transplantation; (6) A follow-up period of ≥0.5 year after the end of treatment (EOT); (7) Absence of HCC development at <0.5 years after the EOT.

The study protocol was approved by the Ethics Committee of Juntendo University Shizuoka Hospital (No. 215, January 2012) and performed in compliance with the Helsinki Declaration (as revised in Brazil, 2013). Each patient gave written informed consent before participating in this study.

### 4.2. Laboratory Investigations and Liver Histology

All routine laboratory data were collected immediately before treatment. The APRI and FIB-4 index was calculated as previously described [19,20]. Serum WFA^+^-M2BP level was measured using the pretreatment serum samples stored at −20 °C. WFA^+^-M2BP quantification was performed by a WFA-antibody immunoassay using a commercially available kit (HISCL M2BPGi; Sysmex Co., Kobe, Japan) and a fully automatic immunoanalyzer (HISCL-5000; Sysmex Co.). SVR was defined as negative for serum HCV RNA at 24 weeks after EOT.

Of the 355 patients, 333 underwent ultrasonography-guided percutaneous liver biopsies just before treatment initiation. Paraffin-embedded liver-biopsy specimens were stained with hematoxylin-eosin, Azan-Mallory, and reticulin silver impregnation, and were evaluated by an experienced pathologist who was blinded to patient-clinical data according to the Metavir classification system [35].

### 4.3. Patient Follow-Up

Examination for serum tumor markers and ultrasonography were performed at least once every six months during the follow-up period. The negativity of serum HCV RNA was reconfirmed annually. HCC diagnosis was confirmed predominantly through imaging studies, including dynamic computed tomography and magnetic resonance imaging. When the lesion was absent for typical imaging features, fine-needle aspiration biopsy was performed. The follow-up period was terminated on 31 March 2016.

### 4.4 Statistical Analyses

Categorical data were compared by the corrected χ-squared method. Continuous variables were analyzed by the Mann–Whitney *U* test. Factors associated with HCC development were determined by the Cox proportional hazard models, and the hazard ratio (HR) and 95% confidence interval (CI) were calculated. The cumulative incidence of HCC development was determined by the Kaplan-Meier method, and differences were tested using the log-rank test. A *p* < 0.05 was considered statistically significant. All statistical analyses were performed using PASW Statistics 18 (IBM SPSS, Chicago, IL, USA).

## 5. Conclusions

Our findings indicate that serum WFA^+^-M2BP level with FIB-4 index could be used to successfully stratify the risk for HCC development in patients achieving SVR and demonstrate the importance of pretreatment WFA^+^-M2BP assessment for the management of patients with CHC.

## Figures and Tables

**Table 1:** Baseline characteristics of patients enrolled in the study.

Characteristics	All Patients (*n* = 355)	With HCC (*n* = 12)	Without HCC (*n* = 343)	*p*-Value
Age, years	56 (20–85)	68 (45–73)	56 (20–85)	0.005 ^‡^
Males, *n* (%)	216 (60.8)	9 (75.0)	207 (60.3)	0.379 ^§^
BMI (kg/m^2^)	23.3 (15.3–39.5)	23.8 (20.2–26.5)	23.3 (15.3–39.5)	0.963 ^‡^
Habitual drinker, *n* (%)	107 (30.1)	3 (25.0)	104 (29.4)	1.000 ^§^
PI use, *n* (%)	56 (15.7)	2 (16.7)	54 (15.7)	1.000 ^‡^
HCV-RNA (logIU/mL) ^†^	6.2 (1.2–7.8)	5.4 (1.2–7.0)	6.2 (1.2–7.8)	0.163 ^‡^
HCV genotype 1, *n* (%)	159 (44.8)	7 (58.3)	152 (44.3)	0.625 ^§^
Stage of fibrosis (F0–2/F3–4) ^†^	288/45	5/6	283/39	0.001 ^§^
Grade of inflammation (A0–1/A2–3) ^†^	113/220	0/11	113/209	0.018 ^§^
Hemoglobin A1_C_ (%)	5.1 (3.4–10.0)	5.1 (4.5–6.7)	5.1 (3.4–10.0)	0.758 ^‡^
Albumin (g/dL)	4.2 (3.3–4.8)	3.8 (3.3–4.3)	4.2 (3.3–4.8)	<0.001 ^‡^
ALT (IU/L)	52 (11–1071)	79 (29–209)	51 (10–1071)	0.168 ^‡^
GGT (IU/L)	36 (8–517)	61 (25–209)	37 (8–517)	0.053 ^‡^
Platelet count (×10^4^/μL)	17.8 (4.3–38.3)	11.4 (7.5–17.9)	18.0 (4.3–38.3)	<0.001 ^‡^
AFP (ng/mL) ^†^	5 (1–870)	12 (3–870)	5 (1–358)	<0.001 ^‡^
FIB-4 index	1.91 (0.31–16.22)	6.10 (2.56–8.93)	1.86 (0.31–16.22)	<0.001 ^‡^
APRI	0.61 (0.14–8.93)	1.93 (0.52–6.17)	0.60 (0.14–8.93)	<0.001 ^‡^

Data are expressed as medians (range) or numbers (%). ^†^ Data not available for all patients; ^‡^ Mann–Whitney *U* test; ^§^ *χ*^2^ test. *p*-values are for comparisons between patients with and without HCC development. HCC, hepatocellular carcinoma; AFP, α-fetoprotein; ALT, alanine aminotransferase; APRI, aspartate aminotransferase-to-platelet ratio index; BMI, body mass index; FIB-4, fibrosis-4; GGT, γ-glutamyl transpeptidase; HCV, hepatitis C virus; PI, protease inhibitor.

**Table 2:** Univariate and multivariate analyses of factors associated with hepatocellular carcinoma development.

**Univariate Analysis**			
**Variables**	**Category**	**HR (95% CI)**	***p*-Value**
Age	<60 years	1	
	≥60 years	8.33 (1.80–38.50)	0.007
Sex	female	1	
	male	1.77 (0.48–6.58)	0.374
BMI	<23 kg/m^2^	1	
	≥23 kg/m^2^	1.72 (0.52–5.74)	0.377
Habitual drinker	No	1	
	Yes	0.93 (0.25–3.45)	0.909
PI use	No	1	
	Yes	1.99 (0.40–9.76)	0.347
HCV-RNA	<6.0 logIU/mL	1	
	≥6.0 logIU/mL	0.24 (0.07–0.90)	0.034
HCV genotype	2	1	
	1	1.56 (0.49–4.92)	0.449
Hemoglobin A1c	<5.5%	1	
	≥5.5%	1.98 (0.59–6.63)	0.270
Albumin	>4.0 g/dL	1	
	≤4.0 g/dL	7.84 (2.34–26.24)	0.001
ALT	<55 IU/L	1	
	≥55 IU/L	3.53 (0.95–13.04)	0.059
GGT	<55 IU/L	1	
	≥55 IU/L	4.40 (1.32–14.69)	0.016
AFP	<8 ng/mL	1	
	≥8 ng/mL	14.35 (3.14–65.60)	0.001
Platelet count	>8.0 × 10^4^ /µL	1	
	≤8.0 × 10^4^ /µL	5.02 (1.08–23.24)	0.039
FIB-4	<3.7	1	
	≥3.7	17.08 (3.69–79.03)	<0.001
APRI	<1.25	1	
	≥1.25	13.85 (2.99–64.09)	0.001
WFA^+^-M2BP	<2.80 COI	1	
	≥2.80 COI	30.43 (3.92–236.04)	0.001
**Multivariate Analysis**			
**Variables**	**Category**	**HR (95% CI)**	***p*-Value**
FIB-4	<3.7	1	
	≥3.7	5.62 (1.14–27.77)	0.034
WFA^+^-M2BP	<2.80 COI	1	
	≥2.80 COI	15.21 (1.77–130.94)	0.013

AFP, α-fetoprotein; ALT, alanine aminotransferase; APRI, aspartate aminotransferase-to-platelet ratio index; BMI, body mass index; CI, confidence interval; COI, cut off index; FIB-4, fibrosis-4; GGT, γ-glutamyl transpeptidase; HCV, hepatitis C virus; HR, hazard ratio; PI, protease inhibitor; WFA^+^-M2BP, *Wisteria floribunda* agglutinin-positive Mac-2-binding protein.

## Abbreviations

AFP	α-Fetoprotein
ALT	Alanine aminotransferase
APRI	Aspartate aminotransferase-to-platelet ratio index
BMI	Body mass index
CHC	Chronic hepatitis C
CI	Confidence interval
COI	Cut off index
DAA	Direct-acting antiviral drug
EOT	End of treatment
FIB-4	Fibrosis-4
GGT	γ-Glutamyl transpeptidase
HCC	Hepatocellular carcinoma
HCV	Hepatitis C virus
HR	Hazard ratio
PI	Protease inhibitor
SVR	Sustained virologic response
WFA^+^-M2BP	*Wisteria floribunda* agglutinin-positive Mac-2-binding protein
